# Comparative Diagnostic Utility of Cell Cytology, Cell Block, Pleural Brushing, and Pleural Biopsy in Exudative Pleural Effusions

**DOI:** 10.7759/cureus.94407

**Published:** 2025-10-12

**Authors:** Peerzada Ajaz A Shah, Mustafa Bashir, Moien Farooq, Tavseef A Tali, Toufeeq A Teli

**Affiliations:** 1 Department of Internal Medicine, Government Medical College (GMC), Baramulla, IND; 2 Department of Internal Medicine, Sher-I-Kashmir Institute of Medical Sciences (SKIMS), Srinagar, IND; 3 Department of Radiation Oncology, Government Medical College (GMC), Baramulla, IND; 4 Department of Internal Medicine, Government Medical College (GMC), Udhampur, IND

**Keywords:** cell block, cytology, exudative pleural effusion, pleural biopsy, pleural brushing

## Abstract

Background: Exudative pleural effusion is a common clinical problem with diverse etiologies, often requiring invasive procedures for definitive diagnosis. While conventional cytology is widely used, its diagnostic yield remains limited. Ancillary methods such as cell block (CB) preparation, pleural brushing, and thoracoscopic pleural biopsy may enhance diagnostic accuracy.

Objectives: This study aimed to evaluate and compare the diagnostic utility of pleural fluid cell cytology (CC), CB, pleural brushing, and pleural biopsy in patients with exudative pleural effusion.

Methods: This prospective observational study was conducted at a tertiary care hospital and included 49 patients with exudative pleural effusion. All patients underwent pleural fluid analysis, cytology, CB preparation, and medical thoracoscopy with pleural brushing and biopsy. Diagnostic yields, sensitivity, specificity, positive predictive value, and negative predictive value of each modality were calculated using pleural biopsy as the reference standard.

Results: The study population had a mean age of 52.4 ± 15.2 years, with 59% of male participants. Malignancy was the most frequent etiology (55%), followed by tuberculosis (28.5%) and nonspecific pleuritis (14.2%). Diagnostic yield was highest for pleural biopsy (83.6%), followed by pleural brushing (80.9%), CB (61.9%), and CC (38.1%). Sensitivity and specificity were 44.4% and 100% for cytology, 59.2% and 100% for CB, and 80.9% and 100% for pleural brushing, respectively. The combination of cytology and CB improved diagnostic yield compared to cytology alone.

Conclusion: Conventional cytology alone has limited sensitivity in diagnosing exudative pleural effusions. CB and pleural brushing significantly improve diagnostic yield, while pleural biopsy remains the gold standard. Incorporating CB and pleural brushing into routine practice may enable earlier and more accurate diagnosis, particularly in suspected malignant effusions.

## Introduction

Pleural effusion refers to the pathological accumulation of fluid within the pleural cavity, typically resulting from either excessive fluid production or impaired lymphatic clearance [1]. It represents the most frequent presentation of pleural pathology, with causes spanning from cardiac and pulmonary diseases and systemic inflammatory disorders to malignancies that necessitate urgent diagnostic evaluation and treatment. Based on underlying mechanisms, pleural effusions are broadly classified as transudative or exudative [2]. Transudates develop when systemic factors influencing pleural fluid dynamics are altered, whereas exudates occur due to structural or functional changes in pleural surfaces or adjacent vasculature. Laboratory analysis of pleural fluid enables this differentiation. Diagnostic thoracentesis is a simple and relatively safe outpatient procedure without absolute contraindications [3]. However, relative contraindications include bleeding disorders, systemic anticoagulation, minimal fluid volume, mechanical ventilation, poor patient cooperation, and cutaneous infections such as herpes zoster at the puncture site [4]. Routine pleural fluid tests include cell counts, pH, protein, glucose, and lactate dehydrogenase (LDH). Additional markers, such as adenosine deaminase (ADA, suggestive of tuberculosis), amylase (for suspected esophageal rupture), N-terminal prohormone of brain natriuretic peptide (NT-pro-BNP, indicating heart failure), and triglycerides (greater than 110 mg/dL in chylothorax), may be helpful [5]. When effusion is detected, establishing its cause is essential. If classified as transudative, further pleural fluid analysis is often unnecessary; however, exudative effusions require additional diagnostic workup to identify the underlying etiology [6]. Categorization of effusion is thus critical to guide differential diagnosis, investigations, and management. Light's criteria remain the standard tool for distinguishing transudates from exudates.

Cytological evaluation of pleural effusions is an established diagnostic modality, with positive findings often sufficient to confirm malignancy and obviate invasive surgical procedures. It is valuable not only for detecting malignant involvement but also for staging and prognostication [7]. However, interpretation can be challenging when differentiating malignant cells from reactive mesothelial cells, which may display atypia due to various insults such as infection, inflammation, or toxic exposure. Similarly, certain tumors like well-differentiated adenocarcinomas may present only subtle cytomorphological changes, further complicating diagnosis. Technical issues related to fixation, staining, or preparation may also affect accuracy [8]. The presence of reactive mesothelial proliferation, excessive inflammatory infiltrates, or inadequate cellular yield is an additional limitation of conventional smears [9]. Diagnostic yield depends on multiple factors, including disease extent, tumor type, and pathologist experience, with published sensitivity rates varying widely between 40% and 90% [10-14].

Cell block (CB) preparation enhances diagnostic accuracy by improving cellular concentration, preserving tissue architecture, and enabling microhistological evaluation [15]. Richardson et al. [16] and Dekker and Bupp [17] reported higher cancer detection rates when CB methods were added to smears. The major advantages include architectural preservation and the ability to cut multiple sections from a single specimen for special stains and immunohistochemistry (IHC) [18]. IHC, increasingly applied to CBs, provides lineage-specific diagnosis and assists in distinguishing adenocarcinoma, squamous cell carcinoma, and small cell carcinoma [19,20]. This method thus enhances accuracy and reproducibility, and reduces the proportion of unclassified malignancies. Combining conventional smears with CBs allows the determination of the primary tumor site with up to 81% accuracy.

Medical thoracoscopy (also known as pleuroscopy) is a highly valuable tool for evaluating exudative pleural effusions, offering a superior diagnostic yield. Pleural biopsy remains the gold standard in unexplained exudative effusions. Unlike video-assisted thoracoscopic surgery, which requires general anesthesia and single-lung ventilation, medical thoracoscopy is usually performed by pulmonologists under local anesthesia with or without conscious sedation [21]. The technique involves inserting a thoracoscope through the chest wall, enabling direct pleural inspection and targeted biopsy. Other modalities such as fluid analysis, blind biopsy, or transthoracic aspiration may fail to establish a diagnosis, highlighting the importance of thoracoscopy. While forceps biopsy is the standard approach, it carries risks such as bleeding and difficulty in sampling lesions on the visceral pleura or near vascular structures. In such cases, pleural brushing under direct thoracoscopic vision provides a safer means of obtaining diagnostic material and can be combined with biopsy to enhance yield [22].

In cases of undetermined pleural effusion, multiple studies support the role of medical thoracoscopy after noninvasive evaluation (history, clinical examination, blood tests, chest imaging, and basic pleural fluid analysis, including cytology, biochemistry, ADA, Gram stain, and acid-fast bacilli (AFB) culture) fails to yield a diagnosis. Most of these studies report diagnostic yields exceeding 80%, often surpassing 90%. Thoracoscopy enables the targeted sampling of abnormal pleura and provides visualization of the visceral pleura, offering additional diagnostic clues. Cytology alone detects metastatic pleural disease in 60%-80% of cases and mesothelioma in fewer than 20% [23]. Therefore, in suspected malignant effusions with an inconclusive initial workup, thoracoscopy with parietal pleural biopsy should be pursued [24]. Since visceral pleural biopsy carries higher risks, most interventional pulmonologists restrict sampling to the parietal pleura. Again, pleural brushing can supplement biopsy, improving the diagnostic yield.

Several studies have compared cytology, CB, brushing, and biopsy in malignant pleural effusions. For instance, Shivakumarswamy et al. [25] observed that CB had a 15% higher diagnostic yield for malignancy compared with cytology alone. Building on this evidence, our study was designed to comprehensively evaluate and compare all four diagnostic modalities, cytology, CB, brushing, and biopsy, in patients with exudative pleural effusion.

## Materials and methods

This study aimed to evaluate the diagnostic utility of pleural fluid cell cytology, CB, pleural brush cytology, and thoracoscopic pleural biopsy in patients with exudative pleural effusions presenting to Sher-I-Kashmir Institute of Medical Sciences (SKIMS).

The present study was designed as a prospective, nonrandomized, observational study conducted in the Department of General Medicine, in collaboration with the Department of Pathology, over 12 months at SKIMS, a tertiary care institute.

Inclusion criteria

The patients whose age was greater than 18 years; hospitalized patients with exudative pleural effusion according to Light’s criteria [26] that remained undiagnosed after detailed history, physical examination, routine investigations (complete blood count, kidney function test, liver function test, blood chemistry, urine examination), chest radiography (X-ray and CT scan), ultrasonography (USG) of chest, and basic pleural fluid analysis including total leucocyte count, differential count, biochemical profile (protein, glucose, LDH), ADA levels for tuberculosis [27,28], Gram stain, AFB smear and culture, and cytology (three samples negative for malignant cells); patients with exudative pleural effusion and cytology positive for malignant cells requiring pleuroscopy for further evaluation and diagnosis; and patients admitted to SKIMS were included in this study.

Exclusion criteria

The patients whose age was less than 18 years; with transudative pleural effusion as per Light’s criteria [26]; who were unfit for thoracoscopy, including unstable cardiovascular or hemodynamic status, with coagulation abnormalities (prothrombin concentration <60% or platelet count <60,000); with absolute contraindications to the procedure, such as obliteration of pleural space by fibrosis or multiloculated effusion; with gross pleural thickening on CT scan impairing lung expansion; and pulmonary arteriovenous aneurysm, suspected hydatid cyst, or highly vascular pulmonary lesions were excluded.

Methodology

Detailed demographic and clinical data (age, sex, occupation, drug history, smoking history, and comorbidities) were recorded. All relevant hematological and radiological investigations were performed.

Patients underwent USG-guided thoracocentesis. Pleural fluid was assessed for physical characteristics (appearance, color, and coagulum) and processed on the same day. At least 50 mL of pleural fluid was aspirated for analysis and divided into three portions: 1) first portion: cytological examination (total and differential leukocyte count, and malignant cell detection), biochemical evaluation (protein, glucose, LDH, and ADA), microbiological tests (Gram stain, culture, and AFB smear and culture); 2) second portion: sent for CB preparation (~20 mL). CBs were prepared by adding 10% alcohol formalin (three times the volume of pleural fluid), centrifuging at 2,000 rpm for 10 minutes, fixing the sediment in alcohol formalin, embedding in paraffin, and processing like small biopsies; and 3) third portion: preserved for additional investigations as clinically indicated, such as NT-pro-BNP for suspected heart failure [29], amylase for suspected esophageal rupture [30], and triglycerides for suspected chylothorax [31]. Pleural fluid cytology was performed by conventional smear preparation after centrifugation at 2,500 rpm for 15 minutes.

Before pleuroscopy, fitness was ensured (age >18 years, no contraindications). The procedure was performed under local anesthesia with an intercostal nerve block. Pleural brushing was carried out at suspected sites (parietal pleura, visceral pleura, or near vascular structures) by scraping suspicious areas multiple times, obtaining greater than or equal to four specimens per patient. This was followed by four to six forceps biopsies from parietal pleural lesions. After the procedure, a 28-32 Fr chest tube was placed, and a chest radiograph was taken.

Forceps biopsy and pleural brush specimens were submitted for histopathological and cytological evaluation. Pleural biopsy tissue was processed in an automatic tissue processor, followed by embedding, block preparation, and sectioning (3-4 μm thickness) for microscopic examination.

Statistical analysis

Statistical analysis was performed using IBM Statistical Package for the Social Sciences Statistics for Windows, version 29.0 (IBM Corp., Armonk, NY). Categorical variables were expressed as frequencies and percentages, while continuous variables were summarized as the mean ± standard deviation or the median with interquartile range, depending on their distribution. Normality of continuous data was assessed using the Shapiro-Wilk test.

Associations between categorical diagnostic test results (cytology, CB, and brushing) and the reference standard (pleural biopsy) were evaluated using the Pearson chi-square test (χ², df = 1); Fisher’s exact test was used when expected cell counts were less than five. Diagnostic accuracy parameters including sensitivity, specificity, positive predictive value (PPV), and negative predictive value (NPV) were calculated with 95% confidence intervals. A p value of <0.05 was considered statistically significant.

This study was conducted in accordance with the Declaration of Helsinki and was approved by the Institutional Ethics Committee of SKIMS in 2022 (approval no. RP 2011/2022). Written informed consent was obtained from all participants before enrolment.

## Results

Baseline characteristics of patients

The study population consisted of 49 patients with a mean age of 52.4 ± 15.2 years. The male population constituted 59%, and the female population 41%. Most patients (73.5%) were from rural areas, while 26.5% belonged to urban areas. About 43% were smokers with a median smoking index of 210 (range 20-320). Common comorbidities included hypertension (32.3%), hypothyroidism (14.3%), and diabetes mellitus (12.2%), while 38% had no comorbidities.

Clinical presentation and radiological findings

Breathlessness (87.8%) was the most frequent presenting symptom, followed by cough (65%) and fever (38%). Right-sided pleural effusion was observed in 29 patients, left-sided in 18, and bilateral effusion in two patients. On CT chest, 31 patients had a massive effusion, 16 had a moderate effusion, and two had a mild effusion. Most effusions were lymphocytic (81.6%), with 18.4% being neutrophilic.

Pleural fluid characteristics

The mean pleural fluid protein was 4.72 ± 1.09 g/dL, the mean ADA was 33.7 ± 36.5 U/L, and the median LDH was 315 U/L. Grossly, 27 patients had straw-colored fluid and 22 had hemorrhagic fluid, as shown in Tables 1, 2.

**Table 1:** Description of pleural fluid parameters LDH: lactate dehydrogenase; SD: standard deviation

Parameter	Statistic	Values
Proteins	Mean ± SD	4.715 ± 1.090 g/dL
Sugar	Median (min-max)	95 (12-213) mg/dL
LDH	Median (min-max)	315 (105-1,860) U/L
Hemorrhagic:straw-colored	Count	22:27

**Table 2:** Distribution of pleural fluid ADA ADA: adenosine deaminase; SD: standard deviation

Diagnosis	Mean ADA (U/L) ± SD
Malignancy	15.8 ± 9.87
Tuberculosis	53.88 ± 59.91
Nonspecific pleuritis	30.25 ± 16.1

Pleural fluid cytology

Cytology was positive for malignancy in 12 patients (24.4%) and negative in 37 patients (75.6%), as shown in Table 3.

**Table 3:** Diagnosis by pleural fluid cell cytology

Diagnosis	Frequency	Percentage
Nonmalignant	37	75.6%
Malignancy	12	24.4%
Total	49	100%

The diagnostic performance compared to biopsy is as follows: sensitivity is 44.4%, specificity is 100%, PPV is 100%, and NPV is 59.5%. The association between cytology and biopsy was statistically significant (χ² = 19.63, p < 0.0001).

Thoracoscopic findings

The predominant finding was pleural nodules/plaques in 36 patients (73.4%). Other findings included hypervascularity/congestion (59.1%), adhesions (28.5%), loculations (16.3%), and normal pleura in two patients (4%).

Pleural brushing

Brushing was performed in 36 patients and was diagnostic in 31 cases. Malignancy was diagnosed in 17 patients (47.2%), granulomas in seven (19.4%), and nonspecific pleuritis in seven (19.4%). It was inconclusive in five patients (13.8%). Diagnostic performance against biopsy is as follows: sensitivity is 80.9%, specificity is 100%, PPV is 100%, and NPV is 78.9%. The association between brushing and biopsy was statistically significant (χ² = 29.74, p < 0.0001).

Pleural biopsy

Pleural biopsy was diagnostic in 48 of 49 patients (98%). Malignancy was the most common diagnosis (27 patients, 55%), followed by tuberculosis (14 patients, 28.5%) and nonspecific pleuritis (seven patients, 14.2%). Only one case (2%) was inconclusive. Among malignancies, adenocarcinoma was most frequent (n = 12), followed by squamous cell carcinoma (n = 6).

Comparison of diagnostic modalities

A head-to-head comparison showed diagnostic yield was highest for pleural biopsy (83.6%), followed by brushing (80.9%), CB (61.9%), and cytology (38.1%), as shown in Table 4.

**Table 4:** Diagnostic yield of different modalities (n = 36 with all four tests done)

Diagnosis	Cell cytology	Cell block	Pleural brushing	Pleural biopsy
Malignant	8	13	17	21
Nonmalignant	28	23	19	15
Diagnostic yield	38.1%	61.9%	80.9%	-
Total (n)	36	36	36	36

The results for the CB analysis are as follows: sensitivity is 59.2%, specificity is 100%, and both the PPV and NPV are recorded at 100% and 66.7%, respectively. Furthermore, the association with biopsy demonstrates a chi-square value of 25.54, with a significance level of p < 0.0001. Cytology + CB combined improved sensitivity compared to cytology alone (Table 5). Pleural brushing had a nearly comparable yield to biopsy.

**Table 5:** Inferential statistics for diagnostic modalities against pleural biopsy PPV: positive predictive value; NPV: negative predictive value

Diagnostic modality	Sensitivity (%)	Specificity (%)	PPV (%)	NPV (%)	χ² value	p value
Cytology	44.4	100	100	59.5	19.63	<0.0001
Cell block	59.2	100	100	66.7	25.54	<0.0001
Pleural brushing	80.9	100	100	78.9	29.74	<0.0001

Complications

Minor complications were observed in 29.6% of patients. Persistent pain (32.6%) was most common, followed by hypoxia (14.3%), fever (10.2%), and subcutaneous emphysema (2%). No major complications or mortality occurred.

## Discussion

Cell cytology

In the cytological evaluation of pleural fluid, 12 patients had a malignant etiology. Compared with the final diagnosis on biopsy, cell cytology had a sensitivity of 44.4% and a specificity of 100% in diagnosing malignant pleural effusions. Previous studies have shown a large variation in sensitivity, ranging from 40% to 90% in diagnosing malignant pleural effusion [10-14]. One such study, conducted by Arnold et al. [32], recruited 921 patients over eight years with exudate pleural effusion and found an overall sensitivity of fluid cytology in diagnosing malignancy of approximately 46%. There was a variation in sensitivity depending on cancer primary, with adenocarcinoma (79%) and ovarian carcinoma (95%) having high pick-up rates compared with mesothelioma (6%) and hematology malignancy (40%). Hirsch et al. [33] recruited 300 patients who required diagnostic thoracentesis. Malignancy was identified as the cause of effusion in 117 (39%) patients. The sensitivity of pleural fluid cell cytology alone to identify malignancy as the cause was diagnostic in 54% of patients. Another study by Prakash and Reiman [34] analyzed pleural effusion in 414 cases and found that pleural fluid cytology was diagnostic in 57.6% of cases with malignant pleural effusion. The results of our study are comparable to those of previous studies, although they have lower sensitivity than some of the previous studies. This could be because not all patients with positive cell cytology were included in the study, as a diagnostic modality other than pleuroscopy was deemed appropriate for further diagnosis. Also, some samples were not processed on the same day of thoracocentesis, which may have led to degeneration of cells and false-negative results. Pleural fluid cytology has high specificity in diagnosing malignant effusions, as also shown by our study. This means that a positive result should be considered a definitive test, which in some cases may obviate the need for further testing.

Cell block

In our study, pleural fluid CB was positive for malignancy in 16 patients out of 49, with a sensitivity of 59.2%, specificity of 100%, NPV of 66.6%, and PPV of 100%. Additionally, the sensitivity of CB (59.25%) was higher than that of cell cytology (44.4%), but the combined sensitivity of both tests was found to be the same (59.25%). The results of our study are very similar to the previous study done by Güldaval et al. [35], where they studied the utility of cell cytology and CB in exudative pleural effusion in 146 patients; out of these, 54 patients were diagnosed as malignant, and 59 patients were diagnosed as benign. The sensitivity, specificity, PPV, and NPV of CB were 59.3%, 100%, 100%, and 72.8%, respectively. Another similar study by Assawasaksakul et al. [36] examined 278 malignant pleural effusions, and they found the sensitivity of CB in diagnosing malignant effusions to be 61.9%, which is comparable to the result of our study (59.2%). Bodele et al. [37] diagnosed an additional 7% malignancies in CB methods when compared with conventional smear methods. In our study, CB provided a diagnosis of malignancy in four more patients (8%) compared to CC.

Pleural brushing

In our study, pleural brushing was done in 36 patients, and malignancy was the most common diagnosis in 17 patients. The sensitivity, specificity, NPV, and PPV of pleural brushing were 80.9%, 100%, 83.3%, and 100%, respectively. The results of our study are comparable to those of Khanduri et al. [38], who presented data from 45 patients with exudative pleural effusion. Pleural brush cytology identified malignancy in 26 patients, 13 with infection, while six samples were deemed inadequate. However, biopsy was positive in 42 cases out of 45 (93.3%) in detecting malignancy and infectious diseases. The sensitivity of pleural brush cytology in malignant pleural effusion is 65.13%, specificity is 73.68%, PPV is 81.48%, and NPV is 77.78%. In our study, the sensitivity was higher because a semirigid thoracoscope BF-1T150 (Olympus Corporation, Tokyo, Japan) was used, which is easy for manipulation and easy to get tissue in difficult areas. However, in Khanduri et al.'s study, a rigid thoracoscope was used. Another study, conducted by Khames et al. [39], found that combined thoracoscopic pleural specimens were diagnostic in 24 patients (96%). Forceps biopsy was positive in 23 patients (92%), while pleural brush was positive in 18 patients (72%). In our study, pleural brushing was positive in 17 patients out of 21 patients (80.9%) with a malignant diagnosis. The results of our study are also comparable to those of a study by Sivagnaname et al. [40], which found that thoracoscopic pleural biopsy was diagnostic in 76 of 80 patients (95%). Thoracoscopic pleural brushing was diagnostic in 74 patients (92.5%). Histopathology revealed malignancy (82.7%), granulomatous inflammation (11.5%), and nonspecific inflammation (5.7%). The sensitivity and specificity of pleural brushing were 96% and 75%, respectively. In our study, a combination of cytology smear and CB was found to have the same sensitivity as CB, i.e., 59.2%, which is in agreement with the study by Güldaval et al. [35]. In another study conducted by Assawasaksakul et al. [36], the sensitivity of CC plus CB was slightly higher (57.2%) than that of cell cytology (48.7%) and CB (49.9%) alone. The reason could be that in our study, the cytopathologists were not blinded to clinical data, and the interpretation of CB results may be influenced by the results of cytology smears. Also, our study found that the combination of cell cytology, CB, and pleural brushings has an overall sensitivity of 80.9%, which is the same as that of pleural brushing (80.9%). There are no previous studies that have examined whether combining these three diagnostic tests in the same patient increases overall sensitivity.

Pleural biopsy

In our study, on pleuroscopic biopsy, the most common diagnosis was malignancy in 27 patients out of 49, followed by tuberculosis in 14 patients. Among malignant pathologies, the most common diagnosis was adenocarcinoma (n = 12), followed by squamous cell carcinoma (n = 6). The diagnostic yield in our study was 83.6%, out of which 27 (55%) were malignant and 14 (28.5%) were tubercular. This finding was comparable to those of most other studies, including Munavvar et al. [41], Wang et al. [42], Blanc et al. [43], Law et al. [44], Diacon et al. [45], and McLean et al. [46]. In eight patients, the cause could not be determined, with seven patients having nonspecific pleuritis and one patient having normal pleura. We did not follow patients who had nonspecific pleuritis or those who had normal pleura, as this was not part of our study. During the postprocedure period, patients were monitored for at least 24 hours. There were no major complications during this observation period. The most common complication was local site pain, followed by hypoxia and fever, which were managed conservatively.

In summary, our study highlights the comparative diagnostic utility of different modalities for exudative pleural effusions. Cell cytology, while highly specific, demonstrated limited sensitivity, indicating that a positive result can often be considered definitive, but negative results require further investigation. CB preparation enhanced the diagnostic yield over cytology alone, preserved tissue architecture, and allowed for additional ancillary studies such as IHC. Pleural brushing showed high sensitivity and specificity, particularly valuable for sampling lesions near vascular structures or on the visceral pleura, offering a safe complement to biopsy. Finally, pleural biopsy reaffirmed its status as the gold standard, providing the highest overall diagnostic yield. Incorporating these insights allows clinicians to strategically combine modalities to maximize diagnostic accuracy and expedite patient management.

Limitations

This study has certain limitations that must be acknowledged. First, the sample size was relatively small (n = 49), which may limit the generalizability of the findings and reduce the statistical power to detect subtle differences between diagnostic modalities. Second, the study was conducted at a single tertiary care center, which may introduce referral bias and restrict external validity. Third, not all patients underwent all four diagnostic modalities; for example, pleural brushing was performed in only 36 patients, potentially affecting direct head-to-head comparisons.

Another limitation is that CC samples were not always processed immediately after collection, which may have contributed to cellular degeneration and false-negative results. Additionally, pathologists were not blinded to clinical data, raising the possibility of observer bias in interpreting cytology and CB specimens. Patients with nonspecific pleuritis or inconclusive diagnoses were not followed up longitudinally, which may have overlooked cases where a definitive etiology could have emerged later. Finally, advanced diagnostic techniques such as IHC and molecular analysis were not routinely incorporated, which could have improved characterization of malignant effusions, especially in cases with equivocal morphology.

## Conclusions

Exudative pleural effusions are commonly encountered in clinical practice. Diagnostic thoracentesis is often inadequate for diagnosing the specific etiology, and invasive procedures, such as pleural brushing and pleural biopsy, significantly increase the diagnostic yield. In our study, in addition to CC, CB has increased the diagnostic yield from 38.09% to 61.90% and to 80.9% by pleural brushing in malignant effusions. Our study has shown that pleural brushing has a good diagnostic yield but has not proven to be better than pleural biopsy. We conclude that the CB technique should be considered as a useful adjuvant in evaluating exudative pleural effusion along with the routine cell cytology method, and pleural brushing can be considered the additional diagnostic technique, especially in sampling pleural lesions close to vascular structure and on visceral pleura and this would help to initiate the treatment early without further waiting for biopsy report. However, due to less number of cases further studies are needed to corroborate with our results.
